# Effects of replacing canola meal with camelina expeller on intake, total tract digestibility, and feeding behavior of beef heifers fed high-concentrate diets

**DOI:** 10.1093/tas/txaa050

**Published:** 2020-04-29

**Authors:** Hèctor Salas, Lorena Castillejos, Cristian Faturi, Alfred Ferret

**Affiliations:** 1 Animal Nutrition and Welfare Service, Department of Animal and Food Sciences, Universitat Autònoma de Barcelona, Bellaterra, Spain; 2 Instituto da Saúde e Produçao Animal, Universidade Federal Rural da Amazônia, Belém, Pará, Brazil

**Keywords:** beef cattle, camelina expeller, canola meal

## Abstract

To assess the effect of inclusion of camelina expeller in beef cattle diets, 24 Simmental heifers were used. Two experiments were carried out. In the first, two free-choice tests, one without and another with molasses, were conducted to know the preference of animals for a total mixed ration (TMR) made with a 90 to 10 concentrate to barley straw ratio, where canola meal (CM) or camelina expeller (CE) was used in the concentrate as a protein source. Heifers were allotted in four pens with two independent feedbunks, one for each diet. In the second, a replicated 4 × 4 Latin square design was used to assess the effects of the replacement of CM with CE on intake, digestibility, and sorting and animal behavior. The experiment was performed in four 28-d periods during which groups of three animals were allotted in each pen of 12.5 m^2^. Diets were formulated with a 90 to 10 concentrate to barley straw ratio and fed as TMR, and they were designed to contain 1) CM as main protein source and 0% of CE (0CE), 2) 3% of CE replacing CM (3CE), 3) 6% of CE replacing CM (6CE), and 4) 9% of CE replacing CM (9CE). In the free-choice test without molasses, heifers showed a greater preference for CM than for CE (38.6 vs. 8.7 kg/d; *P* < 0.001). When molasses were added to the diet, the preference for CM was maintained (39.1 vs. 9.8 kg/d; *P* < 0.001). Dry matter (DM), organic matter (OM), crude protein (CP), and neutral detergent fiber (NDF) intake was unaffected by the level of replacement of CM by CE (*P* > 0.10), and there was no effect of this replacement on DM, OM, CP, and NDF apparent digestibility (*P* > 0.10). Intake of long particle size increased lineally as CE proportion increased (*P* = 0.015). In addition, extension of sorting behavior for long particle size tended to increase lineally (*P* = 0.07), and sorting against this particle size was detected in 0CE and 3CE, but not in 6CE and 9CE (*P* < 0.05). However, the results recorded for long particle size intake and for sorting behavior against these particles did not translate into more time spent ruminating in heifers fed diets with higher proportion of CE. In conclusion, when canola meal was replaced with camelina expeller at more than 14% of inclusion, heifers preferred the canola meal diet. However, replacing canola meal with camelina expeller up to 9% of inclusion in diets for beef cattle did not affect intake and digestibility but promoted a greater intake of long particle size of barley straw.

## INTRODUCTION

Common plant protein sources, such as soybean meal and canola meal, are expensive and subject to volatile prices. These economic circumstances are driving the research of potential new protein sources for beef cattle diets that can reduce ration cost without compromising animal performance. Camelina sativa is a plant with low agronomic requirements and very tolerant to adverse climatic conditions (Putnam et al., 1993). The industry’s interest in using camelina seeds to produce biofuel has generated coproducts that can be useful as protein sources to feed animals. Colombini et al. (2012) studied different genotypes of Camelina sativa and, after biodiesel extraction, stated that they have potential for use in ruminant rations as a high-quality protein source. Basically, two coproducts can be considered: camelina expeller after a mechanical oil extraction, and camelina meal when, in addition to mechanical extraction, a solvent is also involved, raising the price of camelina meal with regard to camelina expeller.

Camelina coproducts have been used in cattle diets for growing heifers (Moriel et al., 2011; Lawrence et al., 2016), dairy cows (Hurtaud and Peyraud, 2007; Halmemies-Beauchet-Filleau et al., 2011), and beef cattle (Cappellozza et al., 2012). Some of these experiments did not detect effects on intake or animal performance (Moriel et al., 2011; Halmemies-Beauchet-Filleau et al., 2011; Lawrence et al., 2016) and concluded that camelina coproducts can replace conventional supplements in the diets. However, others described a significant decrease in dry matter intake (DMI) when a forage diet was supplemented with a limited amount of concentrate containing camelina meal (Cappellozza et al., 2012) or a tendency to a decreased DMI when camelina meal was used in a dairy cow diet (Hurtaud and Peyraud, 2007). When comparing in situ the two camelina coproducts, Salas et al. (2019) concluded that they differ in crude protein (CP) coefficient of degradability, being higher in camelina expeller than in camelina meal. This difference was confirmed when they were included in isoenergetic and isonitrogenous diets based on these protein ingredients, resulting in a different dietary nitrogen flow when tested in an in vitro trial using a dual flow continuous culture system, and with 8 d of incubation (Salas et al., 2019). However, neither camelina expeller nor camelina meal differed from canola meal, which was also included in this in vitro study, shows a similar efficiency of microbial protein synthesis. This similarity would suggest that camelina coproducts could substitute canola meal in beef cattle diets.

The main concern with feeding camelina coproducts is the antinutritional compounds found in all *Brassica* species (Tripathi and Mishra, 2007). Camelina contains glucosinolates and erucic acid (Zubr, 1997). Glucosinolates can induce a lower activity of the thyroid gland and cause metabolism disturbances, and erucic acid induces myocardial lipidosis in rats (Putnam et al., 1993). Glucosinolate content is higher in camelina than in canola meal (20 to 30 mmol/g vs. <10 mmol/g, respectively). However, camelina does not contain the progoitrin that forms the toxic goitrin, which can decrease hormone production in the thyroid, so camelina coproducts are of moderate toxicity with regard to their glucosinolate content (Matthäus, 1997). The content of erucic acid is usually higher in camelina coproducts than in canola meal (2% to 5% vs. <2%, respectively), but much lower than in high-erucic rapeseed oil. Our hypothesis was that considering the lower price of camelina expeller in comparison with camelina meal, the potential nutritive value of camelina expeller and the similarity of chemical characteristics and efficiency of microbial protein synthesis with canola meal, camelina expeller could be considered a protein source in beef cattle diets. Thus, due to the lack of information on the effects of inclusion of camelina expeller on high-concentrate diets for beef cattle, our objective was to study its effects on intake, nutrient digestibility, sorting, and feeding behavior, and to asses whether it could totally or partially substitute canola meal in beef feedlot diets.

## MATERIALS AND METHODS

Two experiments were carried out from October 2017 to February 2018. In the first, two free-choice tests were performed to ascertain if animals showed a preference when they were offered two different diets, one based on canola meal and another on camelina expeller. In the second, we studied the effects of replacing canola meal with camelina expeller on intake, digestibility, and feeding behavior of beef heifers fed high-concentrate diets. Animal procedures were approved by the Institutional Animal Care and Use Committee (reference CEEAH 1676) of the Universitat Autònoma de Barcelona (Spain) in accordance with the European directive 2010/63/EU.

### Free-Choice Tests

Twenty-four Simmental heifers [273 ±9.6 d old and with an average initial body weight (BW) of 258.6 ±3.6 kg] were allotted in four pens, with six heifers per pen. Pen was considered the experimental unit in which the two treatments tested were separately offered at the same time (four replicates per treatment). Body weight in each pen was on average 259.5, 256.1, 258.3, and 261.3 kg. Each pen of 25 m^2^ was equipped with two independent feedbunks located 2.5 m apart and a water trough beside each one. To record feed intake, each feedbunk was mounted on a waterproof digital platform scale (model DI-160; DIGI I’s Ltd, Maesawa-cho, Isawa-gun, Iwake, Japan). Each scale was programmed to transmit the feed weight at 5-s intervals to a computer with appropriate data capture software (LabView; National Instruments Corporation, Austin, TX). Water consumption was recorded with direct reading flow meters (B98.32.50, Invesy model 510 C, Tashia S.L., Artesa de Segre, Spain).

Two isoenergetic (2.8 Mcal ME/kg DM) and isonitrogenous (13% CP, on DM basis) concentrates were formulated to meet recommendations of the Fundación Española para el Desarrollo de la Nutrición Animal (FEDNA, 2008) for beef cattle to obtain a weight gain of 1.2 kg/d. One concentrate was based on canola meal (CM) as main protein source and another on camelina expeller (CE). The main ingredients of the concentrate, except minerals and premix, were ground through a 5-mm screen. Each concentrate was manually mixed with a mechanically chopped barley straw to achieve a 90 to 10 concentrate to straw ratio, and diets were offered ad libitum as total mixed ration (Table 1). After the first free-choice test where CM at 15.8% was replaced with CE at 14.6% of inclusion [on dry matter (DM) basis], a second one was carried out, this time including in both treatment diets 5% of molasses (as-fed basis). Each free-choice test consisted of 9 d in which both diets were simultaneously available. The first 3 d were for adaptation to diets and feedbunks, followed by two consecutive 3-d periods for pen intake control. We swapped over the diet in the two feedbunks every 3 d (including a position change after the adaptation period), aiming to avoid a possible feeder location effect on animal choice. After the first free-choice test, the second one was performed with the inclusion of molasses. Prior to starting the study, heifers were fed a commercial diet where the protein sources tested were not included.

**Table 1.** Ingredients and chemical composition of treatment diets tested in the free-choice tests^1^

	CM^2^	CE
Ingredients, % (DM basis)		
Corn grain	37.5	26.9
Barley grain	25.8	36.6
Canola meal	15.8	—
Camelina expeller	—	14.6
Soybean hulls	7.7	8.9
Barley straw	10.3	10.5
Salt	0.5	0.5
Bicarbonate	1.0	1.0
Calcium carbonate	0.9	0.9
Vitamin–mineral premix^3^	0.4	0.4
Chemical composition, % (DM basis)^4^		
DM	90.0 ± 0.36^5^	90.4 ± 0.34
Ash	4.2 ± 0.18	4.5 ± 0.20
CP	13.3 ± 0.72	13.2 ± 0.79
EE	2.4 ± 0.05	3.4 ± 0.03
NDF	25.0 ± 0.64	25.3 ± 0.82
ADF	13.1 ± 0.48	13.6 ± 0.54
Lignin	1.5 ± 0.09	1.7 ± 0.06
NFC^6^	55.1 ± 0.29	53.6 ± 0.42

^1^Five percent of molasses (as-fed basis) was added to each concentrate using a mixer feeder wagon in the second free-choice test.

^2^CM = Total mixed ration with canola meal as main protein source; CE = Total mixed ration with camelina expeller as main protein source.

^3^Vitamin–mineral premix (Pinsos Nutribó S.A., Sant Joan de les Abadeses, Girona, Spain) contained per kg (as fed): 8,400 IU vitamin A, 1,680 IU vitamin D_3_, 39.0 mg Fe, 0.7 mg I, 0.7 mg Co, 3 mg Cu, 30 mg Mn, 85 mg Zn, 0.2 mg Se, 78 mg calcium propionate, 42 mg malic acid, 187 mg sepiolite, 2 mg of natural extract from *Castanea sativa* and *Vitis vinifera*, 0.1 mg natural extract from *Satureja hortensis*, 9 × 10^9^ cfu of *Saccharomyces cerevisae*.

^4^DM = dry matter; CP = crude protein; EE = ether extract; NDF = neutral detergent fiber; ADF = acid detergent fiber; NFC = nonfibrous carbohydrates.

^5^Mean ± SD.

^6^NFC is calculated as follows: 100 − (CP + ash + NDF + EE).

Feed bunks were cleaned and refusals collected at 0900 h each morning, and feed was offered twice daily at 0930 and 1630 h, increasing the offer by at least 15% of the previous day’s intake in each individual feed bunk. Samples of offered diets of each feeder were collected daily and dried in a forced air oven at 60 °C for 48 h, ground in a hammer mill through a 1-mm screen (P. PRAT SA, Sabadell, Spain) and stored for later chemical analysis. Dry matter content of offered feed was determined by drying samples for 24 h in a forced air oven at 103 °C according to AOAC (1990; ID 950.05). Nitrogen content of offered feed was determined by the Kjeldahl procedure (AOAC, 1990; ID 976.05), and ether extract (EE) was performed according to AOAC (1990; ID 920.30). The neutral detergent fiber (NDF) and acid detergent fiber (ADF) contents of offered diets were determined sequentially by using an Ankom Fiber Analyzer (Ankom Technology, Fairport, NY) in accordance with the methodology provided by the company. This is based on the procedure of Van Soest et al. (1991) using a thermostable α-amylase and sodium sulfite, and expressed on an ash-free basis. The lignin content of offered feed was determined after fiber procedures using 72% sulfuric acid. The allyl isothiocyanate level was determined by a destilation–volumetry procedure according to the European Directive 71/250/EEC. The erucic acid content was analyzed by chromatography (Model 6890, Hewlett Packard, Palo Alto, CA), according to American Oil Chemists’ Society (AOCS) method CE 2–66 (AOCS, 1998).

### Canola Meal Replacement with Camelina Expeller

Twenty-four Simmental heifers (initial full BW of 294.7 ± 3.7 kg) were allotted in eight pens, in groups of three heifers per pen, considering pen the experimental unit. Grouping was made with the aim to have a similar average pen BW. Each pen was randomly assigned to one of four experimental treatments in a 4 × 4 replicated Latin square design. The experiment was performed in four 28-d periods during which groups of three animals were allotted in each pen of 12.5 m^2^, equipped with a feed bunk and a water trough. Measurements were made in groups of three animals because we wanted to control intake and feeding behavior in competitive conditions. In summary, in each period of each Latin square we had one pen per treatment and three heifers per pen. The experiment lasted 112 d. The same previously described system was used to record feed intake and water consumption.

Four isoenergetic (2.8 Mcal ME/kg DM) and isonitrogenous (13% CP, on DM basis) concentrates were formulated to meet Fundación Española para el Desarrollo de la Nutrición Animal (FEDNA, 2008) recommendations for beef cattle to obtain a weight gain of 1.2 kg/d. Diets were designed to contain 1) CM as main protein source and 0% of CE (0CE), 2) 3% of CE replacing CM (3CE), 3) 6% of CE replacing CM (6CE), and 4) 9% of CE replacing CM (9CE). The main ingredients of the concentrate, except minerals and premix, were ground through a 5-mm screen. Each concentrate was manually mixed with mechanically chopped barley straw to achieve a 90 to 10 concentrate to straw ratio, and diets were offered ad libitum as total mixed ration (Table 2).

**Table 2.** Ingredients and chemical composition of treatment diets with camelina expeller (CE) instead of canola meal

	Diets^1^			
	0CE	3CE	6CE	9CE
Ingredients, % (DM basis)				
Corn grain	37.5	35.5	33.6	31.6
Barley grain	25.8	27.8	29.8	31.8
Canola meal	15.8	12.9	9.9	7.0
Camelina expeller	—	2.7	5.4	8.1
Soybean hulls	7.7	8.0	8.2	8.4
Barley straw	10.3	10.3	10.3	10.2
Salt	0.5	0.5	0.5	0.5
Bicarbonate	1.0	1.0	1.0	1.0
Calcium carbonate	0.9	0.9	0.9	0.9
Vitamin–mineral premix^2^	0.4	0.4	0.4	0.4
Chemical composition, % (DM basis)^3^				
DM	90.0 ± 0.37^4^	90.1 ± 0.34	90.1 ± 0.34	90.4 ± 0.44
Ash	4.2 ± 0.17	4.6 ± 0.17	4.2 ± 0.17	4.6 ± 0.15
CP	13.3 ± 0.82	13.1 ± 0.89	13.3 ± 0.78	13.0 ± 0.79
EE	2.4 ± 0.02	2.8 ± 0.04	2.8 ± 0.02	3.0 ± 0.03
NDF	25.0 ± 0.61	25.2 ± 0.69	24.4 ± 0.63	24.7 ± 0.61
ADF	13.1 ± 0.53	13.2 ± 0.69	13.0 ± 0.70	12.9 ± 0.67
Lignin	1.5 ± 0.05	1.6 ± 0.06	1.7 ± 0.06	1.4 ± 0.09
NFC^5^	55.1 ± 0.40	54.3 ± 0.43	55.3 ± 0.39	54.7 ± 0.41
Particle size, %				
Long	5.32	5.59	5.74	5.71
Medium	2.37	2.11	2.26	2.14
Short	1.61	1.56	1.56	1.66
Fine	90.70	90.73	90.45	90.48

^1^0CE = diet with a 0% of CE; 3CE = diet with a 3% of CE; 6CE = diet with a 6% of CE; 9CE = diet with a 9% of CE.

^2^Vitamin–mineral premix (Pinsos Nutribó S.A., Sant Joan de les Abadeses, Girona, Spain) contained per kg (as fed): 8,400 IU vitamin A, 1,680 IU vitamin D_3_, 39.0 mg Fe, 0.7 mg I, 0.7 mg Co, 3 mg Cu, 30 mg Mn, 85 mg Zn, 0.2 mg Se, 78 mg calcium propionate, 42 mg malic acid, 187 mg sepiolite, 2 mg of natural extract from *Castanea sativa* and *Vitis vinifera*, 0.1 mg natural extract from *Satureja hortensis*, 9 × 10^9^ cfu of *Saccharomyces cerevisae*.

^3^DM = dry matter; CP = crude protein; EE = ether extract; NDF = neutral detergent fiber; ADF = acid detergent fiber; NFC = nonfibrous carbohydrates.

^4^Mean ± SD.

^5^NFC is calculated as 100 − (CP + ash + NDF + EE).

Feedbunks were cleaned and refusals collected at 0900 h each morning, and feed was offered twice daily at 0930 and 1630 h, increasing the offer by at least 15% of the previous day’s intake. Samples of feed offered and refusals were collected daily for seven consecutive days and composited for each pen in each sampling week (day 22 to day 28). After collection, samples were dried in a forced air oven at 60 °C for 48 h, ground in a hammer mill through a 1-mm screen (P. PRAT SA, Sabadell, Spain) and stored for later chemical analysis to analyze DM content, chemical composition, and particle size. Chemical determinations were the same as described previously. Particle size separation of offered feed and refusals were performed using the three-screen Penn State Particle Separator (Nasco, Fort Atkinson, WI), obtaining four different fractions: bigger than 19 mm (long); between 8 and 19 mm (medium); between 1.18 and 8 mm (short); and smaller than 1.18 mm (fine). Sorting was calculated as the actual intake of each fraction size expressed as a percentage of the predicted intake, where predicted intake of each fraction equals the product of as-fed intake and as-fed fraction in the diet. Values <100% indicate selective refusals, >100% is preferential consumption, and equal to 100% is no sorting (Leonardi and Armentano, 2003). Full BW of heifers was recorded on two consecutive days at the beginning and end of the experiment and at the end of each experimental period.

Total tract apparent digestibility of treatment diets was estimated using TiO_2_ as external marker. From day 6 to day 18 of each period, 3 kg of a concentrate premix containing 10 g of TiO_2_/kg was mixed with treatment diets and administered daily in each pen, following Russell et al. (2016). From day 16 to day 19 of each period, fecal samples of all animals were collected daily from the rectum at 0730 h before feed administration. To obtain a representative sample of the pen, 50 g of sample from each animal in the same pen was taken and then mixed. Fecal samples were dried in a forced air oven at 60 °C for 48 h, ground in a hammer mill through a 1-mm screen (P. PRAT SA, Sabadell, Spain), and stored for later chemical analysis to analyze DM, organic matter (OM), CP, and NDF content. From the marker concentration of offered feed and feces, DM digestibility of the diets was estimated with the following equation:

$$\begin{array}{ll} DM\;digestibility\;= & \;(1-[TiO_{2}\;in\;of\;fered\;feed]/[TiO_{2}in\;feces])\\ & \times 100 \end{array}$$

The concentration of the marker in samples of offered feed and feces was determined using the procedure outlined by Myers et al. (2004). Duplicate 0.5-g samples were weighed in 250-mL Kjeldahl digestion tubes. Three and a half g of K_2_SO_4_ and 0.4 g of CuSO_4_ and 13 mL of concentrated H_2_SO_4_ were added to each tube. Samples were exposed to a temperature of 420 °C for 2 h. Ten milliliters of 30% H_2_O_2_ was added to each tube. The total liquid weight was brought up to 100 g using distilled water and filtered through Whatman No. 541 filter paper to remove any precipitate. The absorbance was measured at 410 nm in a calibrated spectrophotometer with working standards, prepared by adding 0, 2, 4, 6, 8, and 10 mg of TiO_2_. To estimate the digestibility of nutrient components, the previous formula was multiplied by the quotient between the concentration of the nutrient in the feces and the concentration of the nutrient in the feed offered.

Animal behavior was recorded using a digital video recording device (model VS-101P VioStor NVR; QNAP Systems Inc., Xizhi City, Taipei County, Taiwan). A digital color camera (model VIVOTEK IP7142; VIVOTEK INC., Chung-HO, Taipei County, Taiwan) was set up in front of the feeding area of each pen at a height of 3 m permitting a full view of the pen. An infrared light with photoelectric cells was set up at each end of the paddock to allow video recording at night (λ = 830 nm and 500 W; Dennard 2020; Dennard, Hants, UK). To study behavioral activities, we used only 1 of the 2 Latin squares included in the experiment. The behavior was video-recorded for 24 h on 2 non consecutive days of each sampling week (day 22 to day 28). In accordance with Madruga et al. (2017a), data processing was carried out by a time sampling method at intervals of 5 min. The behavioral activities recorded were eating and ruminating. Data for each activity are presented as the total time, expressed in minutes, in which the animal maintained this specific activity. An observation was recorded as eating when the animal had its muzzle in the feedbunk or was chewing or swallowing food with its head over it. Ruminating included the regurgitation, mastication, and swallowing of the bolus. Eating and ruminating time, expressed as min/kg total DM and min/kg NDF, were calculated taking into account both time spent eating and ruminating and total DM and NDF intake recorded. Time spent masticating resulted from the sum of eating and ruminating activities.

### Statistical Analyses

To analyze the preference between the diets offered in the free-choice test, data were statistically analyzed using a mixed-effects linear regression model from the MIXED procedure of SAS (version 9.3; SAS Institute Inc., Cary, NC). The model contained the fixed effects of treatment and feeder position, the random effect of pen, and day as a repeated measure. To analyze the preference between diets in each hour of the day, a paired *t*-test was used (SAS Inst. Inc.). Pen was considered the experimental unit, and data were expressed as feed intake (as-feed basis) per pen. For the Latin square experimental design, the data for each pen, calculated as an average of the three heifers per pen fed a given treatment diet at each period, were considered the experimental unit in all the analyses. In each experimental period, the daily mean value was calculated as the average either of 7 d for DM and nutrient intake, DM intake by particle size, and sorting behavior, of 3 d for digestibility data, or of 2 d for behavioral activities. The normality of the data was checked with the UNIVARIATE procedure of SAS (v. 9.3; SAS Institute Inc.). All these data were statistically analyzed using a mixed-effects linear regression model from the MIXED procedure of SAS (version 9.3; SAS Institute Inc.). The model contained the fixed effects of Latin square, treatment, and period, and the random effect of pen nested within Latin square. In the case of behavioral activities, the Latin square effect was not considered, and day was included as a repeated measure. Orthogonal contrasts were used to determine the linear and quadratic effects of increasing the proportion of CE in the diet. To determine whether heifers sorted against or for each particle size, sorting behavior was tested for a difference from 100 using a *t*-test. Significance was declared at *P* < 0.05, and tendencies are discussed at *P* < 0.10.

## RESULTS AND DISCUSSION

### Free-Choice Tests

**Figure 1.**
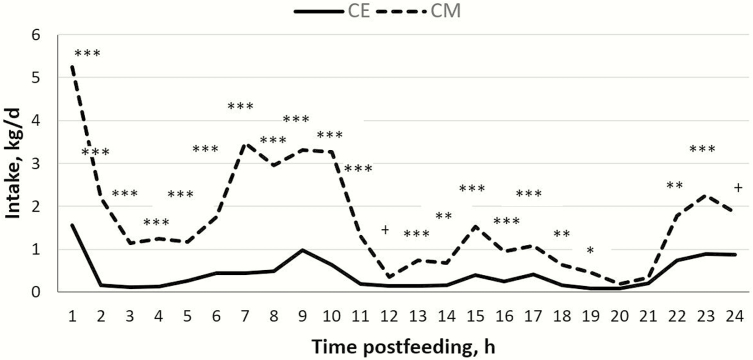
Pen feed intake over the day in free-choice test 1 without molasses added to diets with camelina expeller (CE) or canola meal (CM). Intake differed between diets: ***P < 0.001; **P < 0.01; *P < 0.05; ^+^*P* < 0.10.

**Figure 2.**
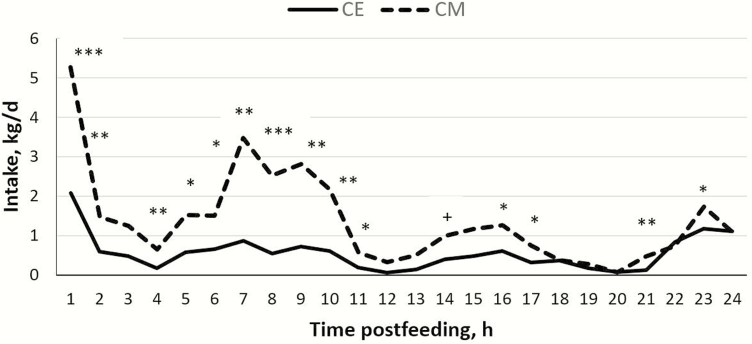
Pen feed intake over the day in free-choice test 2 with molasses added to diets with camelina expeller (CE) or canola meal (CM). Intake differed between diets: ***P < 0.001; **P < 0.01; *P < 0.05; ^+^*P* < 0.10.

Free-choice experiments can be used to assess the willingness of animals to ingest certain experimental feeds when offering different feeds separately at the same time, thus determining their preference for, or the palatability of different feeds (Meier et al., 2012). When the first free-choice test was performed, heifers showed a higher preference for CM than for CE (38.6 vs. 8.7; *P* < 0.001; Table 3), and this preference was maintained throughout the day (Fig. 1). We considered three possible explanations for this result. First, it could have been a case of feed neophobia, in which animals eat only a small amount of a new feed when it is first offered (Provenza, 1995). Low intake in feedlot diets is often observed when animals are initially received into feedlots (Zinn, 1988). However, considering that heifers used in the present experiment did not know either of the two main protein sources used, we discarded this explanation. As a second possibility, we took into account the antinutritional factors, which are very common in all *Brassica* species (Tripathi and Mishra, 2007), and their adverse effects on feed intake (D’Mello, 2000). Although ruminants are more tolerant to glucosinolates than nonruminants, it is recommended not to exceed 10% of inclusion of camelina coproducts in cattle diets (FEDNA, 2010). To discard this possible effect, we analyzed the content of allyl isothiocyanate and erucic acid in both protein ingredients. The resulting amounts were 0.118 and <0.05 mg/g of allyl isothiocyanate and <0.01 and 0.02 g/100 g of erucic acid for CM and CE, respectively. The content of allyl isothiocyanate of CE, as a major metabolite of glucosinolates, was below that of CM, both values being under the range of values (between 0.3 and 2.1 mg/g) obtained by Tripathi and Mishra (2007) in different varieties of CM obtained in diverse oil extraction processes. Values of erucic acid presented by CM and CE were below 1% of the fat fraction considered to be the threshold of CM (EFSA, 2016). Therefore, the content of antinutritional factors suggests that their use would not represent a nutritional problem for heifers. Finally, we considered the problem of palatability, and for this reason, we decided to include in a second free-choice test 5% of molasses to cover up a possible bad taste and/or bad odor. However, heifers continued to express a greater preference for CM than for CE (39.1 vs. 9.8; *P* < 0.001; Table 3), which once again was maintained throughout the day (Fig. 2). Nevertheless, it is not possible to entirely rule out a problem of palatability because 5% of molasses may have been insufficient to make CE diet more palatable for heifers. In both free-choice tests, we did not find a significant effect (*P* > 0.05) of either the position of the pen in the barn or the change of the position of the feedbunk in the pen on feed intake.

**Table 3.** Average as-fed feed intake (kg/pen/d) of heifers fed canola meal and camelina expeller diets in free-choice tests

	Diets^1^			
Item	CM	CE	SEM	*P*-value
Free-choice test 1^2^				
Intake, kg/d	38.6	8.7	0.90	0.001
Free-choice test 2^3^				
Intake, kg/d	39.1	9.8	0.66	0.001

^1^CM = TMR with canola meal as main protein source; CE = TMR with camelina expeller as main protein source.

^2^Free-choice test with diets without molasses.

^3^Free-choice test with diets with molasses.

### Canola Meal Replacement With Camelina Expeller

Dry matter intake and OM intake was unaffected by diet, being on average 8.93 ± 0.214 and 8.53 ± 0.208 kg/d, respectively (*P* > 0.10, Table 4). Because diets were formulated to be isonitrogenous and with similar NDF content, intake of CP and NDF (on average 1.17 ± 0.040 and 2.14 ± 0.054 kg/d, respectively; *P* > 0.10, Table 4) were not different, in accordance with the similar DM and OM intake. Water consumption did not differ among diets (*P* > 0.10, Table 4). The fact that the maximum level of CE inclusion in the concentrate was 9%, unlike the free-choice test where CE was included at 14.6%, and heifers had 3 wk of diet adaptation, could explain the absence of palatability problems. These results contrast with the finding by Cappellozza et al. (2012), working with beef steers fed a diet based on mixed alfalfa-grass hay offered ad libitum and receiving grain-based supplements, that total DMI was reduced when camelina meal was included in the supplement offered (2.04 kg of DM/steer) instead of soybean meal (2.20 kg of DM/steer). These authors included camelina meal at 6.3% (on a DM basis) in one of the experiments carried out and at 8.5% in another. In the present study, CE was included at 2.7% (3CE), 5.4% (6CE), and 8.1% (9CE) in the diet, and DMI was not affected. The main differences between both experiments were that we used CE instead of camelina meal and that we formulated a diet with a 90 to 10 concentrate to straw ratio, while in their experiment, the amount of concentrate was limited, and animals consumed forage ad libitum. In fact, Cappellozza et al. (2012) reported that camelina meal impaired both forage intake and total DMI. In agreement with our results, Lawrence et al. (2016) did not report differences in DMI when growing heifers were fed diets containing 60% grass hay and 40% concentrate mix, in which either 10% camelina meal, 10% distillers dried grains with solubles, or 10% linseed meal was used as an ingredient in the concentrate.

**Table 4.** Intake and water consumption of heifers fed diets with different proportion of canola meal and camelina expeller as main protein source

	Diets^1^					*P*-value	
Item^2^	0CE	3CE	6CE	9CE	SEM	Linear	Quadratic
Intake, kg/d							
DM	8.88	8.90	9.06	8.89	0.214	0.780	0.553
OM	8.47	8.51	8.66	8.49	0.208	0.747	0.488
CP	1.17	1.18	1.16	1.17	0.040	0.819	0.866
NDF	2.10	2.13	2.17	2.14	0.054	0.437	0.424
Water consumption, L/day	26.95	25.40	26.81	24.92	1.120	0.161	0.918

^1^0CE = diet with a 0% of CE; 3CE = diet with a 3% of CE; 6CE = diet with a 6% of CE; 9CE = diet with a 9% of CE.

^2^DM = dry matter; OM = organic matter; CP = crude protein; NDF = neutral detergent fiber.

Total tract apparent digestibility of DM, OM, CP, and NDF was linearly unaffected by diet, but a tendency for a quadratic effect was detected in DM and OM digestibility (*P* = 0.055; Table 5). This unclear quadratic tendency and the nonlinear effect on nutrient digestibilities would confirm those that were obtained in vitro in a previous experiment (Salas et al., 2019) when canola meal and camelina expeller were tested individually as ingredients or when these were included in a diet for beef in proportions similar to those used in the free-choice tests and using a dual flow continuous culture system. In an in vitro experiment, also using fermenters, Brandao et al. (2018) compared the digestibility of three diets with 0, 50, and 100% of a solvent-extracted camelina meal replacing canola meal. They did not detect any effect of diet on DM, OM, CP, and ADF digestibility, but observed a linear decrease in NDF digestibility. These authors suggested that increased inclusion of camelina meal mainly affected hemicellulose digestibility, which they related to the decreased acetate proportion in diets containing camelina meal detected in their experiment. This different result could be related to the type of diet used. Although Brandao et al. (2018) formulated diets for dairy cows that contained 55% hay and 45% concentrate, we designed diets for fattening beef with 10% barley straw and 90% concentrate.

**Table 5.** Total tract apparent digestibility of the diets with different proportions of canola meal and camelina expeller

	Diets^1^					*P*-value	
Item^2^	0CE	3CE	6CE	9CE	SEM	Linear	Quadratic
Apparent digestibility, % DM							
DM	64.76	63.14	62.36	65.33	1.548	0.851	0.055
OM	68.59	67.25	66.97	68.85	1.092	0.883	0.055
CP	54.49	53.99	52.23	53.39	1.860	0.396	0.535
NDF	36.92	32.70	33.61	35.57	2.602	0.702	0.115

^1^0CE = diet with a 0% of CE; 3CE = diet with a 3% of CE; 6CE = diet with a 6% of CE; 9CE = diet with a 9% of CE.

^2^DM = dry matter; OM = organic matter; CP = crude protein; NDF = neutral detergent fiber.

When different particle sizes of diets were separated using the three-screen Penn State Particle Separator, the proportions of each particle size were similar among diets, being on average 5.6 ± 0.08, 2.2 ± 0.05, 1.6 ± 0.02, and 90.6 ± 0.06 % for long, medium, short, and fine particles, respectively (Table 2). However, intake of long particle size increased linearly as CE proportion increased in the diet (Table 6; *P* < 0.015), whereas intake of remaining particle size did not differ among diets. This result corresponds with the tendency for a linear increase of sorting extension for this particle size (*P* = 0.07) and with a sorting behavior against long particle size (Table 7; *P* < 0.05) detected in 0CE and 3CE, but not in 6CE and 9CE, where no sorting was observed (Table 7). In addition, sorting for fine particle size was detected in heifers fed all diets except for 6CE (Table 7), this particle size corresponding essentially with the concentrate ingredient of diets. Sorting behavior has been reported in growing calves (Miller-Cushon et al., 2013; Groen et al., 2015; Gordon and DeVries, 2016) and growing heifers (Greter et al., 2008; DeVries et al., 2014). Specifically, sorting behavior with preferential consumption for concentrate in total mixed ration (TMR) in beef heifers fed high-concentrate diets was described by Madruga et al. (2017b), confirming the result obtained in the present experiment.

**Table 6.** Intake by particle size of heifers fed diets with different proportions of canola meal and camelina expeller

	Diets^1^					*P*-value	
Item	0CE	3CE	6CE	9CE	SEM	Linear	Quadratic
Intake by particle size^2^, kg/d							
Long	0.35	0.44	0.48	0.46	0.043	0.015	0.063
Medium	0.09	0.10	0.11	0.11	0.026	0.674	0.835
Short	0.08	0.08	0.10	0.09	0.017	0.310	0.768
Fine	8.25	8.28	8.38	8.24	0.203	0.901	0.549

^1^0CE = diet with a 0% of CE; 3CE = diet with a 3% of CE; 6CE = diet with a 6% of CE; 9CE = diet with a 9% of CE.

^2^Particle size determined by Penn State Particle Separator.

**Table 7.** Effect of diet on sorting behavior of heifers fed diets with different proportions of canola meal and camelina expeller

	Diets^1^					*P*-value	
Item	0CE	3CE	6CE	9CE	SEM	Linear	Quadratic
Particle size^2^							
Long	78.26* ^3^	87.75*	91.18	90.90	7.138	0.070	0.316
Medium	47.85	54.82	58.65	56.48	12.808	0.450	0.600
Short	56.67	60.36	65.71	62.70	11.304	0.499	0.662
Fine	103.21*	102.56*	102.18	102.46*	0.840	0.295	0.412

^1^0CE = diet with a 0% of CE; 3CE = diet with a 3% of CE; 6CE = diet with a 6% of CE; 9CE = diet with a 9% of CE.

^2^Particle size determined by Penn State Particle Separator.

^3^Values equal to 100% indicate no sorting, <100% selective refusals (sorting against), and >100% preferential consumption (sorting for).

*Statistical differences from 100% are expressed as follows: *P* < 0.05.

Both the greater intake of long particles and the lack of sorting for this particle size recorded in diets with a greater proportion of CE would indicate that heifers fed these diets needed to consume more barley straw. This might suggest that, in accordance with the free-choice tests, animals detected some palatable characteristics that induced them to intake more straw than animals fed 0CE. Considering these results, differences in the chewing activity of heifers fed these diets could be expected. However, it was observed that the small increase in the intake of longer particles was not enough to promote differences in chewing activity. Time spent eating and ruminating, expressed as min/d, min/kg DM, and min/kg NDF, was unaffected by diet (*P* > 0.10; Table 8), and only a numerical linear increase in time spent ruminating and chewing was observed as the CE proportion increased, when expressed as min/kg NDF with a *P*-value of 0.131 and 0.121, respectively.

**Table 8.** Effect of diet on animal behavior of heifers fed diets with different proportions of canola meal and camelina expeller

	Diets^1^					*P*-value	
Item	0CE	3CE	6CE	9CE	S.E.M.	Linear	Quadratic
Eating							
Min/d	95.5	91.8	100.2	91.9	7.27	0.907	0.590
Min/kg DM	10.4	10.0	10.8	10.3	0.55	0.809	0.993
Min/kg NDF	43.6	41.5	45.0	44.1	2.35	0.511	0.715
Ruminating							
Min/d	361.1	369.3	390.2	376.2	21.50	0.381	0.509
Min/kg DM	40.1	40.0	42.0	42.9	2.79	0.254	0.802
Min/kg NDF	167.2	165.6	175.9	183.7	11.91	0.131	0.585
Chewing							
Min/d	456.6	461.1	490.4	468.1	21.69	0.421	0.452
Min/kg DM	50.5	50.0	52.7	53.2	2.94	0.260	0.813
Min/kg NDF	210.8	207.0	220.9	227.8	12.55	0.121	0.558

^1^0CE = diet with a 0% of CE; 3CE = diet with a 3% of CE; 6CE = diet with a 6% of CE; 9CE = diet with a 9% of CE.

In conclusion, when heifers had the opportunity to choose between two isoenergetic and isonitrogenous diets, one with camelina expeller at 14.6% of inclusion and another with canola meal at 15.8% of inclusion (on DM basis), they preferred the canola meal diet. When canola meal was replaced with camelina expeller up to 9% of inclusion, intake and digestibility of these high-concentrate diets offered to beef heifers were unaffected. However, intake of long particle size increased as camelina expeller proportion increased, and heifers fed at 0% and 3% of camelina expeller inclusion sorted against long particle size. The fact that this size corresponds mainly with barley straw, we may surmise that heifers fed diets with camelina expeller as protein source needed to consume more barley straw probably to cover up some palatable characteristics of camelina expeller.
